# SOX18 Promotes the Proliferation of Dermal Papilla Cells via the Wnt/β-Catenin Signaling Pathway

**DOI:** 10.3390/ijms242316672

**Published:** 2023-11-23

**Authors:** Mingliang He, Xiaoyang Lv, Xiukai Cao, Zehu Yuan, Tesfaye Getachew, Yutao Li, Shanhe Wang, Wei Sun

**Affiliations:** 1 College of Animal Science and Technology, Yangzhou University, Yangzhou 225009, China; 2 Joint International Research Laboratory of Agriculture and Agri-Product Safety of Ministry of Education of China, Yangzhou University, Yangzhou 225009, Chinayuanzehu1988@163.com (Z.Y.); 3 International Joint Research Laboratory in Universities of Jiangsu Province of China for Domestic Animal Germplasm Resources and Genetic Improvement, Yangzhou University, Yangzhou 225009, China; 4 International Centre for Agricultural Research in the Dry Areas, Addis Ababa 999047, Ethiopia; 5 CSIRO Agriculture and Food, 306 Carmody Rd, St Lucia, Brisbane, QLD 4067, Australia

**Keywords:** *SOX18*, DPCs, proliferation, Wnt/β-catenin

## Abstract

SRY-box transcription factor 18 (*SOX18*) is known to play a crucial role in the growth and development of hair follicles (HF) in both humans and mice. However, the specific effect of *SOX18* on sheep hair follicles remains largely unknown. In our previous study, we observed that *SOX18* was specifically expressed within dermal papilla cells (DPCs) in ovine hair follicles, leading us to investigate its potential role in the growth of hair follicles in sheep. In the present study, we aimed to examine the effect of *SOX18* in DPCs and preliminarily study its regulatory mechanism through RNA-seq. We initially found that the overexpression of *SOX18* promoted the proliferation of DPCs compared to the negative control group, while the interference of *SOX18* had the opposite effect. To gain further insight into the regulatory mechanism of *SOX18*, we conducted RNA-seq analysis after knocking down *SOX18* in Hu sheep DPCs. The result showed that the Wnt/β-Catenin signaling pathway was involved in the growth process of DPC after *SOX18* knockdown. Subsequently, we investigated the effect of *SOX18* on the Wnt/β-Catenin signaling pathway in DPCs using TOP/FOP-flash, qRT-PCR, and Western blot (WB) analysis. Our data demonstrated that *SOX18* could activate the Wnt/β-Catenin signaling pathway in DPCs. Additionally, we observed that *SOX18* could rescue the proliferation of DPCs after inhibiting the Wnt/β-Catenin signaling pathway. These findings underscore the essential role of *SOX18* as a functional molecule governing the proliferation of DPCs. Additionally, these findings also greatly enhance our understanding of the role of *SOX18* in the proliferation of DPCs and the growth of wool in Hu sheep.

## 1. Introduction

Wool production is a crucial aspect of the sheep breeding industry, as wool serves as an important fiber material in clothing manufacturing and an important economic source in many countries [1]. Consequently, it is indispensable for the steady development of the wool industry to pay attention to wool production. In China, the Hu sheep is esteemed for its production of patterned wool, which is highly favored by consumers. Therefore, enhancing the wool production of Hu sheep holds great significance. The growth of wool emanates from the hair follicle, which serves as a signature of mammalian skin [2]. Hair follicles consist of various epidermal layers, including the hair stroma, medulla, and inner root sheath stratum corneum, as well as two types of dermal tissue, dermal papilla (DP) and connective tissue [3]. These epidermal layers and dermal tissues consist of epithelial cells, HF stem cells and their progenitor cells, matrix cells, mesenchymal cells (DP cells), and melanocytes. These cells cooperate in facilitating the proper growth and development of hair follicles [4,5,6,7,8]. DPCs are essential for guiding HF stem cell progenitors’ proliferation, upward migration, differentiation, and the forming of other types of HF cells [9,10]. Furthermore, studies have shown that DPCs can promote hair follicle growth and affect the size and shape of hair when the number of DPCs reaches a certain level [11,12,13]. Consequently, it is of great significance to investigate the proliferation of DPCs and gain insight into the regulation mechanism that promotes their proliferation.

During the growth and development of hair follicles, multiple signaling pathways have a significant effect. These pathways include the Wnt/β-Catenin, TGF-β/BMP, and FGF signaling pathways, which work together to ensure the normal growth and development of hair follicles [14,15,16,17,18]. Specifically, the Wnt/β-Catenin signaling pathway also plays a crucial role in DPCs. Previous studies have shown that fish oil could enhance hair growth and DPC proliferation by activating the Wnt/β-Catenin pathway [19]. *Wnt10b*, one of the Wnt family members, could promote DPC proliferation via the Wnt/β-Catenin pathway [20]. These findings highlight the importance of the Wnt/β-Catenin pathway in DPCs and emphasize the need for understanding its role in Hu sheep DPCs.

*SOX18* is a member of the SOX family, which is composed of a class of SRY- (sex determination region of Y-chromosome) related genes with a highly conserved HMG-box DNA-binding domain. These genes encode SOX (SRY-related HMG-box) transcription factors that play a role in regulating embryonic development and cell fate determination [21]. Studies have shown that mutations in *SOX18* lead to the development of oligotrichosis-lymphedema-telangiectasia (HLT) syndrome in humans, which is also responsible for severe cardiovascular and hair follicle defects in ragged (RA) mice [22,23]. RA heterozygotes display ragged hair in RA mice, whereas homozygotes are completely hairless and have a lower survival rate [24]. Through gene targeting, DAVID et al., created *SOX18*^-/-^mice, which displayed only a slight fur defect and a reduced proportion of serrated hair but did not have significant cardiovascular defects [25]. In addition, it has been discovered that hair defects in RA mice are caused by a decrease in the number of hair follicles [26]. In Hu sheep, there are two wool phenotypes: curly and straight, but the cause of the phenotypic difference is still unknown. In previous research, we identified that *SOX18* expression was specific in DPCs using 10× genomics single-cell sequencing on the hair follicles of Hu sheep with curly and straight wool phenotypes [27]. Based on this finding, we hypothesized that *SOX18* plays a crucial role in DPCs. There are limited reports on *SOX18* in sheep DPCs, and the effect of *SOX18* remains unknown. In addition, it is also still unclear whether *SOX18* can affect the DPCs of Hu sheep by regulating the Wnt/β-catenin pathway.

In this study, we aim to investigate the effect of *SOX18* on DPCs. Our findings demonstrate that *SOX18* has a positive effect on the proliferation of DPCs by activating the Wnt/β-catenin pathway. These results will contribute to a better understanding of the mechanisms underlying DPC proliferation and wool growth in Hu sheep.

## 2. Results

### 2.1. SOX18 Is Differentially Expressed in Hu Sheep Skin and DPCs between Different Wool Phenotypes

**Figure 1:**
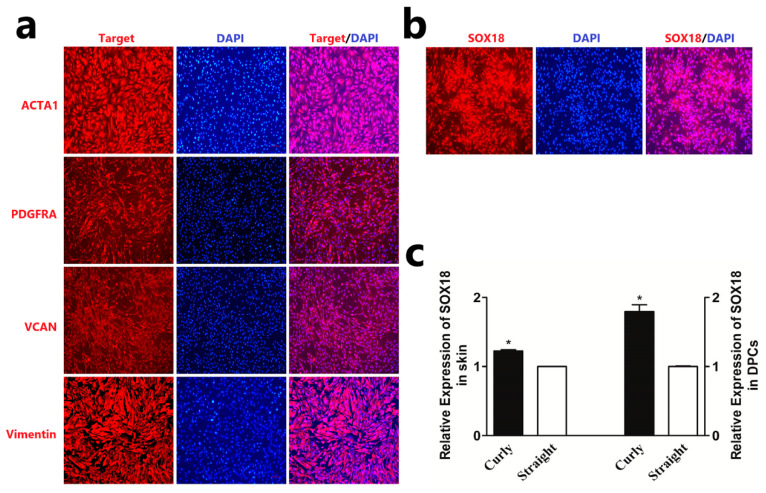
Differential expression of *SOX18* in different wool phenotypes. (**a**) The expression of marker genes in Hu sheep DPCs, the scale is 100 µm. (**b**) The expression of *SOX18* in Hu sheep DPCs, the scale is 100 µm. (**c**) Differential expression of *SOX18* in Hu sheep skin and DPCs. The data are presented as means ± SEM (standard error of the mean) (*n* = 3). The statistical significance was assessed using the unpaired Student’s *t*-test. (* *p* < 0.05).

In our previous study, we discovered that *SOX18* is specifically expressed in the dermal papilla cells of hair follicles [27]. Additionally, it has also been reported to play an essential role in the hair formation of humans and mice [22,23,24,25]. These findings suggest that *SOX18* may be crucial in the formation of Hu sheep wool. Initially, we screened two specific expression genes of DPCs, *PDGFRA*, and *VCAN* from single-cell RNA-seq results for the identification of DPCs [27]. Additionally, we selected two known dermal cell genes, *α-SMA* (*ACTA1*) [28] and *Vimentin* [29], as positive controls. Immunofluorescence staining demonstrated the high purity of the extracted DPCs (Figure 1a). Subsequently, immunofluorescence staining results showed that *SOX18* was expressed in DPCs (Figure 1b). Furthermore, we also observed that *SOX18* was expressed in hair follicle tissue [30]. Next, we assessed the expression of *SOX18* in the skin of Hu sheep with varying wool phenotypes. The results indicated that *SOX18* was highly expressed in the skin of Hu sheep with curly wool phenotypes compared to those with straight wool phenotypes (Figure 1c). Additionally, we found that *SOX18* was highly expressed in the DPCs isolated from the skin of Hu sheep with curly wool phenotypes (Figure 1c). These findings suggest that the gene *SOX18* is likely a crucial gene in the formation of wool in Hu sheep.

### 2.2. SOX18 Promotes DPC Proliferation

**Figure 2:**
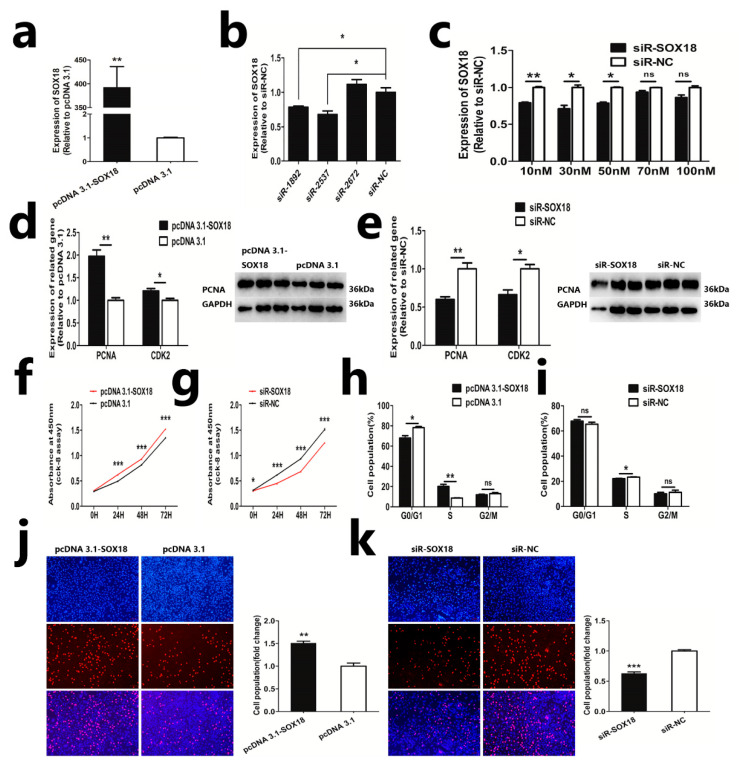
*SOX18* promotes DPC proliferation. (**a**) Detection of mRNA level of *SOX18* following overexpression of *SOX18* in DPCs. (**b**,**c**) Detection of mRNA level of *SOX18* following knockdown of *SOX18* in DPCs. (**d**,**e**) Detection of mRNA level of *PCNA*, *CDK2*, and protein level of *PCNA* following overexpression or knockdown of *SOX18* in DPCs. (**f**,**g**) CCK-8 assay following overexpression or knockdown of *SOX18* in DPCs, respectively. (**h**,**i**) Cell cycle assay following overexpression or knockdown of *SOX18* in DPCs, respectively. (**j**,**k**) EdU assay following overexpression or knockdown of *SOX18* in DPCs, respectively. The scale is 100 µm. The data are presented as means ± SEM (standard error of the mean) (*n* = 3). The statistical significance was assessed using the unpaired Student’s *t*-test. (ns: *p* > 0.05; * *p* < 0.05; ** *p* < 0.01; *** *p* < 0.001).

To investigate the effect of *SOX18*, we constructed an overexpression vector and transfected it into DPCs. As a result, we observed a significant increase in the mRNA expression of *SOX18* in DPCs (Figure 2a). In addition, we designed and synthesized three small interfering RNA sequences and evaluated their effects on *SOX18* expression in DPCs. Our qRT-PCR analysis indicated that the siR-2537 sequence at a concentration of 30 nM was the most effective at reducing *SOX18* expression (Figure 2b,c). Then we detected the effect of *SOX18* on DPCs using qRT-PCR, WB, Cell Counting Kit-8 (CCK-8), 5-ethynyl-2’-deoxyuridine (EdU), and Flow Cytometry. The result of qRT-PCR and WB demonstrated that the overexpression of *SOX18* significantly increases the mRNA level of *PCNA*, *CDK2*, as well as the protein level of *PCNA*. Conversely, the knockdown of *SOX18* resulted in the opposite effects (Figure 2d,e). Furthermore, the CCK-8 assay and EdU assay revealed that overexpression of *SOX18* facilitated cell vitality and proliferation, while knockdown of *SOX18* suppressed these processes, respectively (Figure 2f,j,g,k). Additionally, our cell cycle assay demonstrated that *SOX18* influenced the proliferation of DPCs by modulating the progression of the G0/G1 phase and S phase in the cell cycle (Figure 2h,i). Overall, these findings indicate that *SOX18* plays a positive role in regulating the proliferation of DPCs in vitro.

### 2.3. SOX18 Regulates the Expression of Downstream Genes

**Figure 3:**
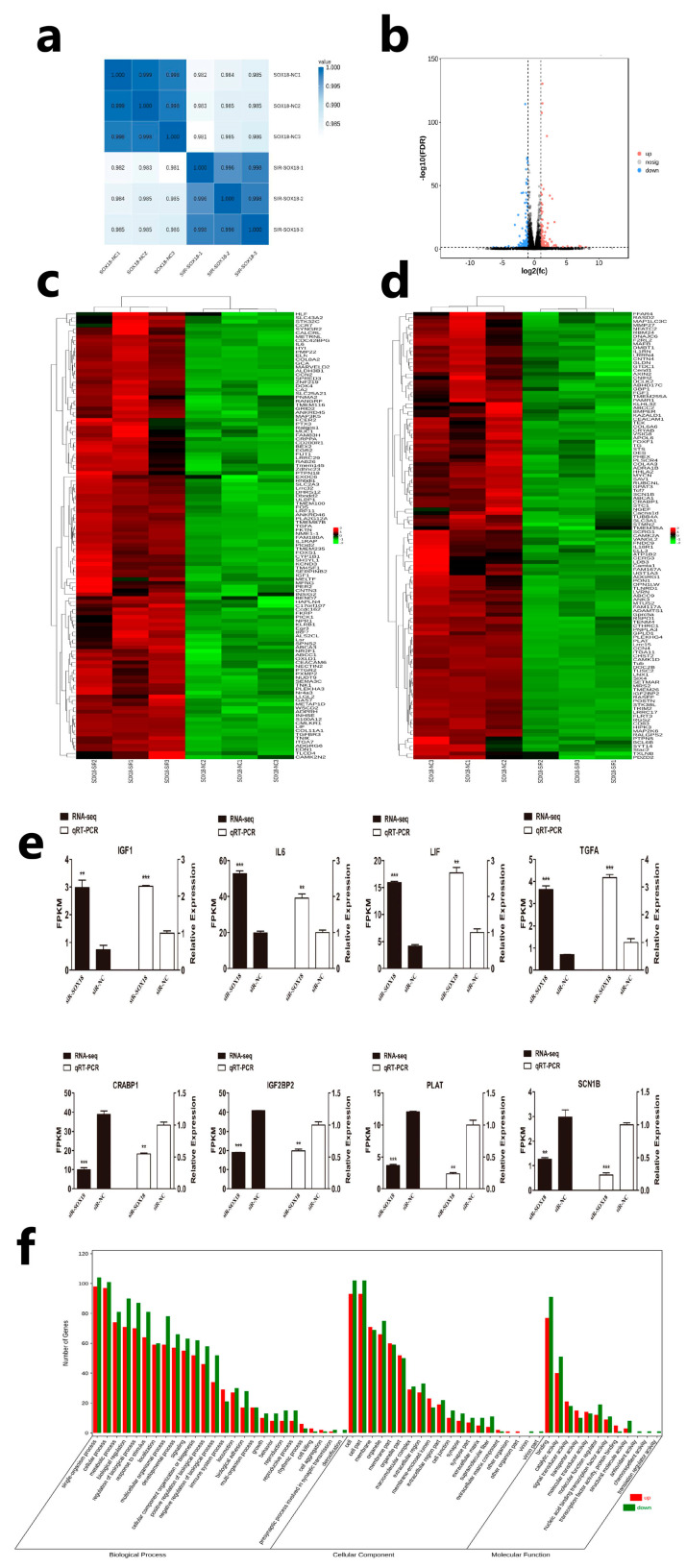
*SOX18* regulates the expression of downstream genes. (**a**) Correlation analysis of samples. (**b**) A volcano plot was used to show the DEGs. (**c**,**d**) Heat map showing expression patterns of significant expression genes. (**e**) Verification of DEGs by QRT-PCR. (**f**) GO enrichment analysis of DEGs. The data are presented as means ± SEM (standard error of the mean) (*n* = 3). The statistical significance was assessed using the unpaired Student’s *t*-test. (** *p* < 0.01; *** *p* < 0.001).

To further investigate the molecular mechanisms of *SOX18* in regulating DPC proliferation, we performed RNA-seq after the knockdown of *SOX18* in DPCs. After completing the quality control for sequencing, Q20, Q30, GC content, and the ratio of clean reads were evaluated for each sample. The RNA sequencing data are shown in Table S1. The sequencing reads were aligned to the sheep reference genome, and the efficiency of aligning the total reads from the six samples to the reference genome exceeded 97%. Over 93% of the reads were aligned to the unique location of the reference genome (Table S1). These results indicated that the data was of high quality and could be used for further analysis. Furthermore, correlation analysis revealed a high degree of similarity among replicate samples within the same group and the RNA-seq data was of a high quality for further analysis (Figure 3a). To identify DEGs in the two groups (siR-SOX18 vs. siR-NC), all the genes sequenced were screened for their difference. The screening process employed the following criteria: a Fold Change ≥ 2 and a False Discovery Rate (FDR) < 0.05. A total of 246 differentially expressed genes (DEGs) were identified, of which 120 were upregulated and 126 were downregulated (Table S2). To directly observe the difference in expression levels of genes and the statistical significance of the differences, a volcano plot was used (Figure 3b). We conducted hierarchical clustering on 120 upregulated genes and 126 downregulated genes. The analysis revealed that samples from the same group exhibited close clustering, further indicating that the sequencing data is accurate and reliable (Figure 3c,d). In addition, we randomly selected four upregulated genes and four downregulated genes for differential expression validation, and the result of qRT-PCR showed the expression trend of eight genes was consistent with the sequencing results (Figure 3e). All of these results indicated that the RNA seq data could be used for the next step of the analysis. Then, we performed a GO analysis, and the results indicated that these DEGs were associated with multicellular organismal processes, regulation of biological processes, nucleic acid binding transcription factor activity, transcription factor activity, and protein binding (Figure 3f; Table S3).

### 2.4. SOX18 Enhances the Wnt/β-Catenin Pathway in DPCs

**Figure 4:**
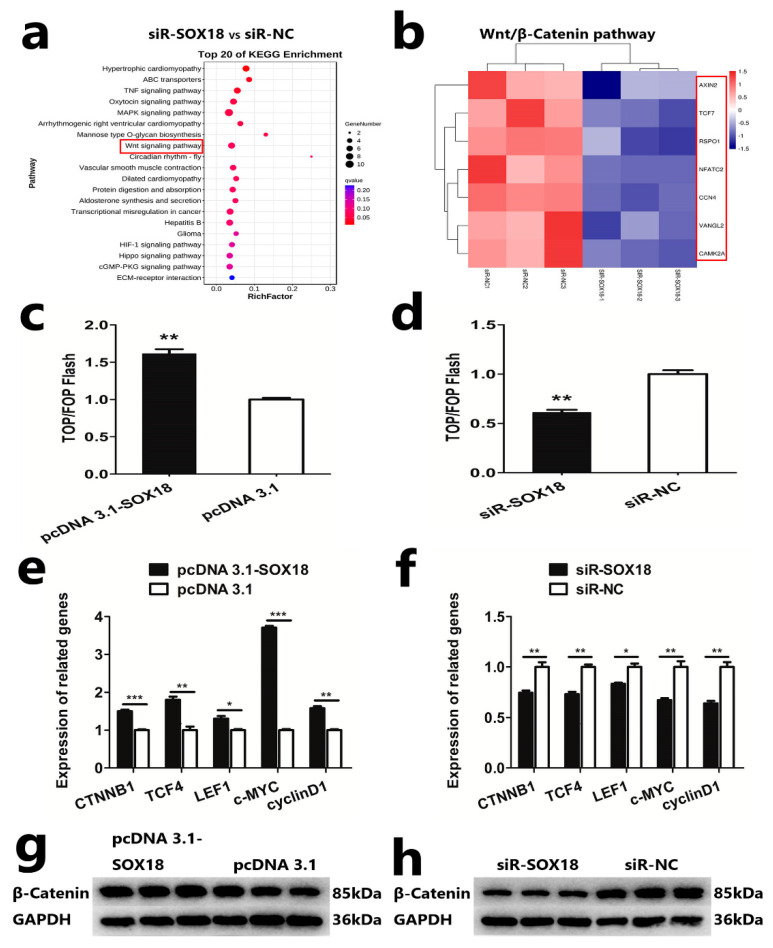
*SOX18* enhances the Wnt/β-Catenin pathway in DPCs. (**a**) KEGG enrichment analysis of DEGs obtained after *SOX18* knockdown. (**b**) DEGs in the siR-SOX18 and siR-NC related to the Wnt/β-Catenin pathway are depicted as a heatmap. The red box indicates upregulation, and the blue box indicates downregulation. (**c**,**d**) TOP/FOP-flash assays following overexpression or knockdown of *SOX18* in DPCs, respectively. (**e**,**f**) Detection of mRNA level of Wnt/β-catenin pathway-related genes following overexpression or knockdown of *SOX18* in DPCs, respectively. (**g**,**h**) Detection of the protein expression of β-Catenin following overexpression or knockdown of *SOX18* in DPCs, respectively. The data are presented as means ± SEM (standard error of the mean) (*n* = 3). The statistical significance was assessed using the unpaired Student’s *t*-test. (* *p* < 0.05; ** *p* < 0.01; *** *p* < 0.001).

Based on the result of the KEGG enrichment analysis of the DEGs obtained from RNA-seq, we identified seven differentially expressed genes (DEGs) that were enriched in the Wnt/β-Catenin pathway (Figure 4a,b; Table S3). This finding prompted us to explore the effect of *SOX18* on the Wnt/β-Catenin signaling pathway. To explore the effect of *SOX18* on the Wnt/β-Catenin pathway, we estimated the transcription activity of β-catenin/TCF in DPCs after overexpression or knockdown of *SOX18* in sheep DPCs, respectively. The results of the dual-luciferase detection indicated that *SOX18* could increase the activity of the Wnt/β-Catenin signaling pathway in DPCs (Figure 4c,d). We also analyzed the mRNA expression level of Wnt/β-catenin pathway-related genes (*CTNNB1*, *TCF4*, *LEF1*, *c-MYC*, and *cyclinD1*) in DPCs. The qRT-PCR assay revealed that *SOX18* could enhance the expression of these genes (Figure 4e,f). Furthermore, we analyzed the protein expression of β-Catenin following the overexpression or knockdown of *SOX18* in DPCs, respectively. The Western blot assay indicated *SOX18* could enhance the protein expression of β-Catenin in DPCs (Figure 4g,h). All of these results suggested that *SOX18* could activate the Wnt/β-catenin pathway in the DPCs of Hu sheep.

### 2.5. SOX18 Regulates DPC Proliferation via the Wnt/β-Catenin Pathway

**Figure 5:**
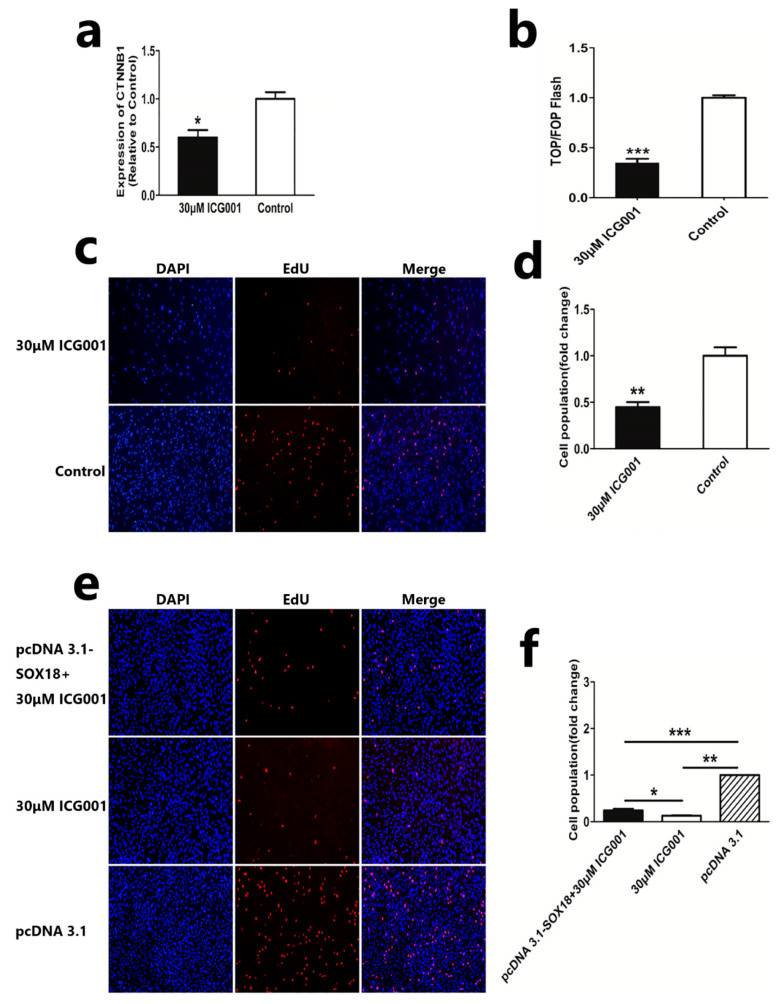
*SOX18* regulates DPC proliferation via the Wnt/β-Catenin pathway. (**a**) Detection of mRNA level of *CTNNB1* following the inhibiting of the Wnt/β-Catenin pathway in DPCs. (**b**) TOP/FOP-flash assays of the Wnt/β-Catenin signaling pathway activity in DPCs treated with/without 30 µM ICG001. (**c**,**d**) EdU assay of DPCs treated with/without 30 µM ICG001. The scale is 100 µm. (**e**,**f**) EdU assay after DPCs were treated with pcDNA 3.1-SOX18 + 30 µM ICG001, 30 µM ICG001, and pcDNA 3.1. The scale is 100 µm. The data are presented as means ± SEM (standard error of the mean) (*n* = 3). The statistical significance was assessed using the unpaired Student’s *t*-test. (* *p* < 0.05; ** *p* < 0.01; *** *p* < 0.001).

Due to the involvement of *SOX18* in the Wnt/β-catenin pathway in DPCs, we aimed to investigate whether *SOX18* promotes the proliferation of DPCs by upregulating the Wnt/β-catenin pathway. First, we detected the mRNA expression of *CTNNB1* after the DPCs were treated with 30 µM ICG001 (Beyotime, Shanghai, China) and found that the expression of *CTNNB1* was significantly reduced compared to the control group (Figure 5a). Next, we estimated the β-catenin/TCF transcription activity in DPCs treated with/without 30 µM ICG001. The results of a dual-luciferase detection indicated that 30 µM ICG001 significantly reduced the activity of the Wnt/β-Catenin signaling pathway in DPCs (Figure 5b). These results indicate that 30 µM ICG001 could inhibit the activity of the Wnt/β-catenin pathway in DPCs. Furthermore, we found that 30 µM ICG001 could reduce the proliferation of DPCs, which reveals the positive effect of the Wnt/β-catenin pathway on the proliferation of DPCs (Figure 5c,d). Then, we investigated whether overexpressing *SOX18* could rescue the proliferation of DPCs after DPCs were treated with 30 µM ICG001. EdU assay indicated that overexpression of *SOX18* could significantly rescue the proliferation of DPCs treated with 30 µM ICG001 (Figure 5e,f). All of these results prompt us to suggest that *SOX18* regulates DPC proliferation through the Wnt/β-catenin pathway.

## 3. Discussion

As an important cell type in hair follicles, DPCs have been reported to play an important role in regulating hair follicle morphogenesis [31]. In addition, DPCs have been found could provide signal transduction and nutrient supply for hair follicle growth and differentiation processes [32]. Therefore, analyzing the growth regulation of DPCs helps us to understand the growth and development of hair follicles. In a previous study, we discovered that the expression of *SOX18* was restricted to DPCs, as opposed to other cell types within the hair follicles [27]. This result makes us question whether *SOX18* plays a role in DPCs growth. It was reported that the dominant-negative mutation of *SOX18* could inhibit the formation and differentiation of DP; this result suggests that *SOX18* plays a role in the dermal papilla and further promotes our research on the proliferation of dermal papilla cells regulated by *SOX18* [33]. Furthermore, *SOX18* has also been found to influence hair growth in humans and mice [22,23]. Therefore, we speculated that *SOX18* might affect the growth of Hu sheep wool by regulating the proliferation of DPCs. In the present study, we detected the effect of *SOX18* on the proliferation of DPCs, and we found that overexpression of *SOX18* could enhance the proliferation of DPCs, while the interference of *SOX18* had the opposite effect. These findings indicate a positive role of *SOX18* in the in vitro proliferation of DPCs.

In a previous study of the cashmere fineness of Jiangnan cashmere goats by RNA-seq, *SOX18* was found to be related to hair follicle development [34]. Thus, in this study, we further understood the function of *SOX18* in DPCs and hair follicles by RNA-seq. Based on the analysis of these differential genes from RNA-seq, we have a preliminary understanding of the potential function of *SOX18* in DPC proliferation and hair follicle development. Based on GO enrichment analysis, we found that some DEGs are related to the cell proliferation process, such as PMP22 [35], CCN2 [36], TCF7 [37], and FOXF1 [38], and these genes were reported to regulate the proliferation of different cells. Therefore, we speculated that *SOX18* affects the proliferation of DPCs by affecting the expression of these genes. Moreover, the result of the KEGG enrichment analysis indicated that DEGs clustered on the TNF signaling pathway [39], MAPK signaling pathway [40], and Wnt signaling pathway [41], and that these signaling pathways also play an essential role in the process of cell proliferation. This result also prompts us to suggest that *SOX18* may affect cell proliferation through these signaling pathways.

According to the result of the pathway enrichment analysis, we found that some genes were related to the Wnt pathway. This discovery drew our attention to the relationship between *SOX18* and the Wnt pathway in DPC. In hair growth, many signal pathways have been discovered to play a crucial role, and the Wnt signaling pathway is of great importance. The Wnt/β-Catenin signaling pathway has been reported to participate broadly in the morphogenesis and periodic growth of hair follicles [42]. In the initiation and regeneration of mice hair follicles, Wnt/β-Catenin signaling has also been proven necessary [43]. In addition, the role of some WNT proteins in hair growth has also been investigated and reported on. Wnt10b, a member of the Wnt family of proteins, could promote dermal papilla cell proliferation and hair follicle growth through the Wnt/β-Catenin pathway [20]. Based on these results, we speculated that *SOX18* may affect the proliferation of DPCs and hair follicle growth through the Wnt/β-Catenin pathway. Thus, we detected the effect of *SOX18* on the Wnt/β-Catenin pathway. We found that several components of the Wnt signaling pathway, including β-Catenin, TCF/LEF, and downstream targets such as c-Myc and cyclinD1, are regulated by *SOX18*. Thereby, we also demonstrated that *SOX18* may regulate the proliferation of DPCs through the activation of the Wnt/β-Catenin pathway.

The Wnt/β-catenin signaling pathway is highly conserved and has been found to participate in the regulation of cell proliferation, differentiation, apoptosis, and many other life activities [44]. The Wnt/β-Catenin signaling pathway plays a critical role in the proliferation of goat DPCs, and similar effects have also been found in sheep DPCs [45,46]. Our study revealed that the promotional effect of *SOX18* on the Wnt/β-Catenin pathway was diminished upon the inhibition of the Wnt/β-catenin pathway in DPCs. Furthermore, the overexpression of *SOX18* could rescue cell proliferation after inhibiting the Wnt/β-Catenin pathway in DPCs. These findings prompt us to suggest that the promoting effect of *SOX18* on DPC proliferation is achieved by regulating the Wnt/β-Catenin pathway.

## 4. Conclusions

In this study, we initially examined the effect of *SOX18* on dermal papilla cells and revealed that *SOX18* positively influences the proliferation of DPCs. Combining RNA-seq and experimental verification, we further revealed that *SOX18* could affect the proliferation of DPCs by influencing the Wnt/β-Catenin pathway. Our study identified the effect of *SOX18* on the proliferation of DPCs in Hu sheep, providing a deeper understanding of the involvement of *SOX18* in the growth of wool in Hu sheep.

## 5. Materials and Methods

### 5.1. Animals and Ethics Statement

The skin tissue used for cell isolation was obtained from a healthy 3-day-old lamb of Hu sheep and was provided by Suzhou Sheep Farm (Suzhou, China). The animal experimental protocols were designed in strict accordance with the “Jiangsu Province laboratory animal management measures”, and received approval from the Animal Ethics Committee of Yangzhou University (Approval number: No. 202103279).

### 5.2. Cell Isolation, Culture, and Transfection

Isolation of DPCs: The skin tissue used for dermal papilla cell isolation was collected from a healthy 3-day-old lamb of a Hu sheep, and the development of hair follicles was in the growth stage. Every effort was made to minimize the pain of the lamb during the collection process and the wound was disinfected until full recovery. We first excised the skin tissue and isolated individual hair follicles after obtaining the skin tissue. Then we obtained the bulged ends of the hair follicles and utilized a tweezer to disrupt the bulged ends, promoting the migration of internal cells. Subsequently, we implanted 2–3 bulged ends of the hair follicles into a 12-well cell culture dish for about a week to obtain DPCs.

Culture of DPCs: the medium consisted of DMEM-F12 (Sigma-Aldrich, St. Louis, MO, USA) with added 10% fetal bovine serum (Gibco, Grand Island, NY, USA), and 1% penicillin-streptomycin-amphotericin (Solarbio, Beijing, China). All the cells utilized in this study were cultured within a cell incubator (Thermo, Waltham, MA, USA) with 5% CO_2_ at 37 °C.

Transfection of DPCs: We transfected cells utilizing the jetPRIME Transfection Reagent (Polyplus, Illkirch, France) and followed the manufacturer’s protocol for cell transfection procedures.

### 5.3. Total RNA Extraction, cDNA Synthesis, Primer Design, and qRT-PCR

We cultured DPCs using a 12-well cell culture dish for the RNA extraction and the density of cell transfection was about 50%. Trizol (Takara, Dalian, China) was used for the total RNA extraction of DPCs. A one-step reverse transcription Kit (Tiagen, Beijing, China) was used to synthesize cDNA. For gene expression level detection, 2 × TSINGKE^®^ Master qPCR mix (Tsingke, Nanjing, China) was employed. The GAPDH gene of sheep was employed as a reference gene. Three repeated tests were conducted for each sample. The relative expression of genes was calculated using the 2^−ΔΔCT^ method [47]. Gene primers were designed using Premier Primer 5.0 software (Premier Biosoft International, Palo Alto, CA, USA). All of these primers were synthesized by Tsingke (Nanjing, China), and their details are provided in Table 1.

### 5.4. RNA Oligonucleotides and Plasmid Construction

To construct the overexpression vector of *SOX18*, we used PrimeSTAR^®^ Max DNA Polymerase (Takara, Dalian, China) to amplify the complete coding domain sequence (CDS) of the *SOX18* gene. Then, the CDS fragment was cloned into the pcDNA 3.1+ reporter vector and HindⅢ and BamHI were the restriction sites. Moreover, we performed sequencing of the recombinant plasmid to confirm the successful insertion of the fragment. The primer is provided in Table 2.

In this study, we utilized small interfering RNAs (siRNAs) to silence the expression of *SOX18*. Both the siRNAs for knocking down *SOX18* and the siRNA negative control (NC) were designed and synthesized by GenePharma (Suzhou, China). The specific oligonucleotide sequences are provided in Table 3.

### 5.5. Immunofluorescence

DPCs were cultured in a 24-well cell culture dish for immunofluorescence. The density of cell transfection was about 30%. First, we performed a washing process on DPCs using 1X PBS (Solarbio, Beijing, China) after the cells reached an appropriate density. The cells were fixed by a 4% paraformaldehyde solution (Solarbio, Beijing, China) and permeated by 0.5% Triton X-100 (Solarbio, Beijing, China). Next, the cells were incubated with 5% BSA (Solarbio, Beijing, China). Finally, the primary antibody was used for incubating the cells in darkness at 4 °C. The following day, 1X PBST (Solarbio, Beijing, China) was used to rinse cells which were subsequently incubated at 37 °C with the secondary antibody. The nuclei of the cells were stained by DAPI (Beyotime, Shanghai, China). We utilized an inverted fluorescence microscope (Nikon, Tokyo, Japan) to observe and capture images of the stained cells. The primary antibodies and their respective dilution ratios are presented in the following: ACTA1 rabbit polyclonal antibody (Sangon Biotech, Shanghai, China, 1:400, D121592), PDGFRA rabbit monoclonal antibody (Abcam, Cambridge, UK, 1:400, ab203491), VCAN rabbit polyclonal antibody (Santa Cruz Biotech, Santa Cruz, The Republic Bolivia, 1:400, D223532), Vimentin mouse monoclonal antibody (Santa Cruz Biotech, Santa Cruz, The Republic Bolivia, 1:400, sc-6260), SOX18 antibody (Bioss, Beijing, China, 1:400, bs-17135R). The secondary antibodies and their respective dilution ratios are presented in the following: Goat Anti-Rabbit IgG H&L (Alexa Fluor^®^ 555) (Abcam, Cambridge, UK, 1:400, Ab150078), Goat Anti-Mouse IgG H&L (Alexa Fluor^®^555) (Abcam, Cambridge, UK, 1:400, Ab150114).

### 5.6. CCK-8 Assay

DPCs were cultured in a 96-well cell culture dish for the CCK-8 assay. The density of cell transfection was about 30%. We detected the cell viability at 0 h (12 h after DPCs transfected), 24 h, 48 h, and 72 h using a CCK-8 Kit (Vazyme, Nanjing, China). The absorbance at 450 nm was detected using a microplate reader (EnSpire, Perkin Elmer, Waltham, MA, USA).

### 5.7. EdU Assay

DPCs were cultured in a 24-well cell culture dish for the EdU assay. The density of cell transfection was about 50%. After 48 h of transfection, we fixed cells for 30 min using 4% paraformaldehyde (Solarbio, Beijing, China). Then Triton X-100 was used to make cell permeabilization. Finally, according to the instruction manual, an EdU Apollo In Vitro Imaging Kit (RiboBio, Guangzhou, China) was used to conduct cell treatment. We utilized an inverted fluorescence microscope (Nikon, Tokyo, Japan) to observe and capture images of the stained cells and Image Pro Plus 6.0 software (Media Cybernetics, Rockville, MD, USA) to analyze images.

### 5.8. Cell Cycle Assay

DPCs were cultured in a 6-well cell culture dish for the cell cycle assay. The density of cell transfection was about 50%. After 48 h of transfection, we collected DPCs using trypsin (Solarbio, Beijing, China). Then, we used 70% ethanol to fix cells overnight at 4 °C. Finally, we used propidium iodide (50 ug/mL, Solarbio, Beijing, China) containing RNaseA (50 ug/mL, TianGen, Beijing, China) to stain cells and incubate them in a dark environment at a temperature of 37 °C for 30 min. After DPCs were processed, we used a FACSAria SORP flow cytometer (BD company, Franklin, NJ, USA) to analyze the number of cells at different stages. Data analysis was performed using ModFit LT 5.0 software (Verity Software House, Bedford, MA, USA).

### 5.9. TOP/FOP-Flash Wnt Report Assays

DPCs were cultured in a 12-well cell culture dish for the TOP/FOP-flash Wnt report assays. The density of cell transfection was about 50%. We utilized the TOP/FOP-flash plasmid (Beyotime, Shanghai, China) to estimate the transcription activity of β-catenin/TCF in DPCs. First, we co-transfected pcDNA 3.1-SOX18/pcDNA 3.1, TOP/FOP-flash plasmid, and the Renilla luciferase reporter plasmid (pRL-TK) into DPCs, respectively. After 48 h of transfection, we utilized a dual-luciferase detection kit (Vazyme, Nanjing, China) to process the cells. Next, we employed a microplate reader (EnSpire, Perkin Elmer, Waltham, MA, USA) to estimate the Luciferase activity.

### 5.10. Total Protein Extraction and Western Blot Assay

DPCs were cultured in a 12-well cell culture dish for the collection of protein samples. The density of cell transfection was about 50%. After 48 h of transfection, cells were collected from each well of the 6-well cell culture dish and treated as one sample, respectively. Then, 150 μL RIPA cell lysates (Beyotime, Shanghai, China) and 1.5 μL protease inhibitors (Beyotime, Shanghai, China) were added to each cell lysate to collect proteins. We employed the BCA Protein Quantification Kit (Vazyme, Nanjing, China) to determine protein concentration. For each protein sample, we added 15 μg for the corresponding protein detection. After the protein sample was denatured, we used 10% polyacrylamide gel electrophoresis to obtain the target protein. The voltage was 80 v when the sample underwent electrophoresis in concentrated gel. After the sample entered the separation gel, the electrophoresis voltage was 120 v. Then, we transferred the target protein to the PVDF membrane (Solarbio, Beijing, China) using the Western blot transfer instrument (BIO-RAD, Hercules, CA, USA). The conditions of protein transfer were 25 v voltage, 1A current, and 15 min. Next, the PVDF membrane was incubated with 5% skimmed milk powder and the primary antibody overnight at 4 °C, respectively. The following day, 1X TBST (Solarbio, Beijing, China) was used to rinse the PVDF membrane and was subsequently incubated with the secondary antibody. After detecting PCNA protein expression, we used a stripping buffer (Beyotime Biotechnology, China) to remove the primary and secondary antibodies. Then, 1X TBST (Solarbio, Beijing, China) was used to rinse the PVDF membrane. Subsequently, we blocked and incubated the PVDF membrane with the antibodies for subsequent experiments. Electrochemiluminescence (ECL) (Beyotime, Shanghai, China) was used for the imprinting display of the PVDF membrane. The ChemDocTMTouch Imaging System (Bio-Rad, Hercules, CA, USA) was used to measure protein expression level. The primary antibodies and their respective dilution ratios are presented in the following: PCNA rabbit polyclonal antibody (Affinity Biosciences, Cincinnati, OH, USA, 1:1000, AF0239), β-catenin mouse monoclonal antibody (Beyotime, Shanghai, China, 1:1000, AF0069), and GAPDH mouse monoclonal antibody (proteintech, Wuhan, China, 1:5000, 60004-1-Ig). The secondary antibodies and their respective dilution ratios are presented in the following: Goat Anti-Rabbit IgG H&L(HRP) (Abcam, Cambridge, UK, 1:5000, ab6721), Goat Anti-Mouse IgG H&L(HRP) (Abcam, Cambridge, UK, 1:5000, ab205719).

### 5.11. RNA-Seq Analysis

DPCs used for RNA-seq were previously isolated and preserved by our research group. We cultured DPCs using six 10 cm cell culture dishes for the collection of RNA-seq samples. Each treatment group cultured DPCs in three 10 cm cell culture dishes, resulting in three RNA-seq samples. After 48 h of transfection, cells were collected as six RNA-seq samples for total RNA extraction, respectively. The total RNA was extracted using the Trizol reagent kit (Invitrogen, Carlsbad, CA, USA). The quality of RNA was assessed using the Agilent 2100 Bioanalyzer (Agilent Technologies, Palo Alto, CA, USA), and confirmed through RNase-free agarose gel electrophoresis. Then, Oligo(dT) beads were utilized for eukaryotic mRNA enrichment. The enriched mRNA was fragmented into short fragments using a fragmentation buffer and then reversely transcribed into cDNA using the NEB Next Ultra RNA Library Prep Kit for Illumina (NEB #7530, New England Biolabs, Ipswich, MA, USA). The purified double-stranded cDNA fragments were end-repaired, and an A base was added and ligated to Illumina sequencing adapters. The ligation reaction was purified using the AMPure XP Beads (1.0X). Ligated fragments were subjected to size selection by agarose gel electrophoresis and polymerase chain reaction (PCR) amplification. The resulting cDNA library was sequenced using Illumina Novaseq6000 by Gene Denovo Biotechnology Co. (Guangzhou, China). After acquiring the raw reads from the sequencing machines, fastp [48] (version 0.18.0) was used to filter out low-quality reads and obtain high-quality clean reads. Additionally, the short-read alignment tool Bowtie2 [49] (version 2.2.8) was used to map reads against the ribosome RNA (rRNA) database and subsequently eliminate rRNA-mapped reads. The remaining clean reads were then used in all subsequent analyses. HISAT [50] software (version 2.2.4) was used for each sample to map paired-end clean reads to the reference genome, which was the latest sheep reference genome (GCF_016772045.1_ARS-UI_Ramb_v2.0).

RNA differential expression analysis of the two groups (SIR-SOX18 vs. SOX18-NC) was performed by DESeq2 [51] software (version 1.42.0), and edgeR [52] (version 4.0.2) was used to analyze RNA differential expression between the two samples. The genes/transcripts with false discovery rate (FDR) parameters below 0.05 and absolute fold change ≥2 were considered differentially expressed genes/transcripts. To gain insights into the functionality of these differentially expressed genes (DEGs) in the SIR-SOX18 vs. SOX18-NC group (which consisted of 246 genes), the Database for Annotation, Visualization, and Integrated Discovery (DAVID) [53] was employed for Gene Ontology [54] functional annotation and KEGG [55] pathway analysis. 

### 5.12. Statistical Analysis

The SPSS 25.0 software (SPSS Inc., Chicago, IL, USA) was conducted for statistical analysis. The unpaired Student’s *t*-test was used for the two-group comparison analysis. The data were considered statistically significant only when *p* < 0.05 (*), *p* < 0.01 (**), or *p* < 0.001 (***). Each experiment included three biological replications. All experiment data are reported as means ± SEM (standard error of the mean).

## Figures and Tables

**Table 1:** Primers used for qRT-PCR.

Gene	Primer Sequence (5′–3′)	Product Size (bp)	Annealing Temperature (°C)	Accession Number
*SOX18*	F: TGTGGGCGAAGGACGAGC R: GCCAAGCCTGGGAGGAGGAG	253	60	XM_027976914.2
*PCNA*	F: CGAGGGCTTCGACACTTAC	97	60	XM_004014340.5
	R: GTCTTCATTGCCAGCACATT			
*CDK2*	F: AGAAGTGGCTGCATCACAAG R: TCTCAGAATCTCCAGGGAATAG	92	60	NM_001142509.1
*CTNNB1*	F: GAGGACAAGCCACAGGATTAT	101	60	NM_001308590.1
	R: CCAAGATCAGCGGTCTCATT			
*TCF4*	F: AACCCTTTCGCCCACCAA	299	60	XM_012103768.4
	R: CAGGCTGATTCATCCCAC			
*LEF1*	F: CAGGTGGTGTTGGACAGATAA	179	60	XM_042251146.1
	R: ATGAGGGATGCCAGTTGTG			
*c-MYC*	F: CCCTACCCGCTCAACGACA	295	60	NM_001009426.1
	R: GGCTGTGAGGAGGTTTGC			
*cyclinD1*	F: CCGAGGAGAACAAGCAGATC	91	60	XM_027959928.2
	R: GAGGGTGGGTTGGAAATG			
*IGF1*	F: TGTGCTTGCTCGCCTTCA	216	60	XM_027965760.2
	R: AGTACATCTCCAGCCTCCTCA			
*IL6*	F: CCTGGTGATGACTTCTGC	361	60	NM_001009392.1
	R: AGTTTCCTGATTTCCCTC			
*LIF*	F: AGTGCCAACAGCCTCTTTATC	337	60	XM_004017472.5
	R: GGCCGTAGGTCACATCCA			
*TGFA*	F: CAGCTTCCCACAGTCAGTTC	318	60	XM_027966972.2
	R: AGCAAGCAGTCCTTCCCT			
*CRABP1*	F: TCGGAGAAGGCTTTGAGG	160	60	XM_027966972.2
	R: AGGATGAGTTCGTCGTTGG			
*IGF2BP2*	F: TGTTGGTGCCATCATCGG	314	60	XM_042233496.1
	R: TTATCTTGGTCCCTGTCTCA			
*PLAT*	F: GGGGACTGCTACACTGGAAA	322	60	XM_012106011.3
	R: TGATGTCGGCGAAGAGGC			
*SCN1B*	F: AGAAGGGCACAGAGGAGTTT	207	60	XM_004015635.5
	R: CGAAGAAGAGCAGGCGGTA			
*GAPDH*	F: TCTCAAGGGCATTCTAGGCTAC	151	60	NM_001190390.1
	R: GCCGAATTCATTGTCGTACCAG			

**Table 2:** Primers used for vector construction.

Primer Name	Primer Sequence (5′–3′)	Product Size (bp)	Annealing Temperature (°C)
OE-SOX18	F: CTAGCGTTTAAACTTAAGCTT ATGCAGAGATCGCCGCTCG	1176	62
	R: CCACACTGGACTAGTGGATCCCTATCCAGAGATGCAGGCGCTG		

**Table 3:** Oligonucleotide sequences for siRNA.

Fragment Name	Sequence (5′–3′)
siRNA-1892	GCAAGGCAUGGAAGGAGCUTT
	AGCUCCUUCCAUGCCUUGCTT
siRNA-2537	ACCAGUACCUCAACUGCAGTT
	CUGCAGUUGAGGUACUGGUTT
siRNA-2672	GCUCUGCUGUCUACUACAGTT
	CUGUAGUAGACAGCAGAGCTT
siRNA-NC	UUCUCCGAACGUGUCACGUTT
	ACGUGACACGUUCGGAGAATT

## Data Availability

The data analyzed in this study are available from the corresponding author upon request.

## Supplementary Materials

The following supporting information can be downloaded at: https://www.mdpi.com/article/10.3390/ijms242316672/s1

## References

[1] Rogers G.E. (1990). Improvement of wool production through genetic engineering. Trends Biotechnol..

[2] Houschyar K.S., Borrelli M.R., Tapking C., Popp D., Puladi B., Ooms M., Chelliah M.P., Rein S., Pförringer D., Thor D. (2020). Molecular Mechanisms of Hair Growth and Regeneration: Current Understanding and Novel Paradigms. Dermatology.

[3] Dai B., Sha R.N., Yuan J.L., Liu D.J. (2021). Multiple potential roles of thymosin beta4 in the growth and development of hair follicles. J. Cell Mol. Med..

[4] Cotsarelis G., Sun T.-T., Lavker R.M. (1990). Label-retaining cells reside in the bulge area of pilosebaceous unit: Implications for follicular stem cells, hair cycle, and skin carcinogenesis. Cell.

[5] Yang H., Adam R.C., Ge Y., Hua Z.L., Fuchs E. (2017). Epithelial-Mesenchymal Micro-niches Govern Stem Cell Lineage Choices. Cell.

[6] Clavel C., Grisanti L., Zemla R., Rezza A., Barros R., Sennett R., Mazloom A.R., Chung C.-Y., Cai X., Cai C.-L. (2012). Sox2 in the dermal papilla niche controls hair growth by fine-tuning BMP signaling in differentiating hair shaft progenitors. Dev. Cell.

[7] Saxena N., Mok K.-W., Rendl M. (2019). An updated classification of hair follicle morphogenesis. Exp. Dermatol..

[8] Slominski A., Wortsman J., Płonka P.M., Schallreuter K.U., Paus R., Tobin D.J. (2005). Hair follicle pigmentation. J. Investig. Dermatol..

[9] Sequeira I., Nicolas J.F. (2012). Redefining the structure of the hair follicle by 3D clonal analysis. Development.

[10] Legué E., Nicolas J.-F. (2005). Hair follicle renewal: Organization of stem cells in the matrix and the role of stereotyped lineages and behaviors. Development.

[11] Stenn K.S., Paus R., Chuong C.-M., Randall V.A., Widelitz R.B., Wu P., Jiang T.-X., Paul M.J., George N.T., Zucker I. (2001). Controls of hair follicle cycling. Physiol. Rev..

[12] Chi W., Wu E., Morgan B.A. (2013). Dermal papilla cell number specifies hair size, shape and cycling and its reduction causes follicular decline. Development.

[13] Rahmani W., Abbasi S., Hagner A., Raharjo E., Kumar R., Hotta A., Magness S., Metzger D., Biernaskie J. (2014). Hair follicle dermal stem cells regenerate the dermal sheath, repopulate the dermal papilla, and modulate hair type. Dev. Cell.

[14] Zhao B., Li J., Zhang X., Dai Y., Yang N., Bao Z., Chen Y., Wu X. (2022). Exosomal miRNA-181a-5p from the cells of the hair follicle dermal papilla promotes the hair follicle growth and development via the Wnt/beta-catenin signaling pathway. Int. J. Biol. Macromol..

[15] Nan W., Li G., Si H., Lou Y., Wang D., Guo R., Zhang H. (2020). All-trans-retinoic acid inhibits mink hair follicle growth via inhibiting proliferation and inducing apoptosis of dermal papilla cells through TGF-beta2/Smad2/3 pathway. Acta Histochem..

[16] Botchkarev V.A., Sharov A.A. (2004). BMP signaling in the control of skin development and hair follicle growth. Differentiation.

[17] Han M., Li C., Zhang C., Song C., Xu Q., Liu Q., Guo J., Sun Y. (2022). Single-cell transcriptomics reveals the natural product Shi-Bi-Man promotes hair regeneration by activating the FGF pathway in dermal papilla cells. Phytomedicine.

[18] Rendl M., Polak L., Fuchs E. (2008). BMP signaling in dermal papilla cells is required for their hair follicle-inductive properties. Genes Dev..

[19] Kang J.-I., Yoon H.-S., Kim S.M., Park J.E., Hyun Y.J., Ko A., Ahn Y.-S., Koh Y.S., Hyun J.W., Yoo E.-S. (2018). Mackerel-Derived Fermented Fish Oil Promotes Hair Growth by Anagen-Stimulating Pathways. Int. J. Mol. Sci..

[20] Wu Z., Zhu Y., Liu H., Liu G., Li F. (2020). Wnt10b promotes hair follicles growth and dermal papilla cells proliferation via Wnt/beta-Catenin signaling pathway in Rex rabbits. Biosci. Rep..

[21] Bowles J., Schepers G., Koopman P. (2000). Phylogeny of the SOX family of developmental transcription factors based on sequence and structural indicators. Dev. Biol..

[22] Cermenati S., Moleri S., Cimbro S., Corti P., Del Giacco L., Amodeo R., Dejana E., Koopman P., Cotelli F., Beltrame M. (2008). Sox18 and Sox7 play redundant roles in vascular development. Blood.

[23] Pennisi D., Gardner J., Chambers D., Hosking B., Peters J., Muscat G., Abbott C., Koopman P. (2000). Mutations in Sox18 underlie cardiovascular and hair follicle defects in ragged mice. Nat. Genet..

[24] Carter T.C., Phillips J.S. (1954). Ragged, a semidominant coat texture mutant. J. Hered..

[25] Pennisi D., Bowles J., Nagy A., Muscat G., Koopman P. (2000). Mice null for *Sox18* are viable and display a mild coat defect. Mol. Cell Biol..

[26] Slee J. (1957). The morphology and development of ragged—A mutant affecting the skin and hair of the house mouse II. Genetics, Embryology and Gross Juvenile Morphology. J. Genet..

[27] Wang S., Wu T., Sun J., Li Y., Yuan Z., Sun W. (2021). Single-Cell Transcriptomics Reveals the Molecular Anatomy of Sheep Hair Follicle Heterogeneity and Wool Curvature. Front. Cell Dev. Biol..

[28] Jahoda C.A.B., Reynolds A.J., Chaponnier C., Forester J.C., Gabbiani G. (1991). Smooth muscle α-actin is a marker for hair follicle dermis in vivo and in vitro. J. Cell Sci..

[29] Ge W., Zhang W., Zhang Y., Zheng Y., Li F., Wang S., Liu J., Tan S., Yan Z., Wang L. (2021). A Single-cell Transcriptome Atlas of Cashmere Goat Hair Follicle Morphogenesis. Genom. Proteom. Bioinform..

[30] Wang S., Hu T., He M., Gu Y., Cao X., Yuan Z., Lv X., Getachew T., Quan K., Sun W. (2023). Defining ovine dermal papilla cell markers and identifying key signaling pathways regulating its intrinsic properties. Front. Veter. Sci..

[31] Yue Z., Liu M., Zhang B., Li F., Li C., Chen X., Li F., Liu L. (2023). Vitamin A regulates dermal papilla cell proliferation and apoptosis under heat stress via IGF1 and Wnt10b signaling. Ecotoxicol. Environ. Saf..

[32] Driskell R.R., Clavel C., Rendl M., Watt F.M. (2011). Hair follicle dermal papilla cells at a glance. J. Cell Sci..

[33] Villani R., Hodgson S., Legrand J., Greaney J., Wong H.Y., Pichol-Thievend C., Adolphe C., Wainwight B., Francois M., Khosrotehrani K. (2017). Dominant-negative *Sox18* function inhibits dermal papilla maturation and differentiation in all murine hair types. Development.

[34] Wu C., Li J., Xu X., Xu Q., Qin C., Liu G., Wei C., Zhang G., Tian K., Fu X. (2022). Effect of the FA2H Gene on cashmere fineness of Jiangnan cashmere goats based on transcriptome sequencing. BMC Genom..

[35] Hou J., Zhuo H., Chen X., Cheng J., Zheng W., Zhong M., Cai J. (2020). MiR-139-5p negatively regulates PMP22 to repress cell proliferation by targeting the NF-kappaB signaling pathway in gastric cancer. Int. J. Biol. Sci..

[36] Kidd M., Modlin I.M., Eick G.N., Camp R.L., Mane S.M. (2007). Role of CCN2/CTGF in the proliferation of Mastomys enterochromaffin-like cells and gastric carcinoid development. Am. J. Physiol. Gastrointest Liver Physiol..

[37] Liu Z., Liu J., Chen T., Wang Y., Shi A., Li K., Li X., Qiu B., Zheng L., Zhao L. (2022). Wnt-TCF7-SOX9 axis promotes cholangiocarcinoma proliferation and pemigatinib resistance in a FGF7-FGFR2 autocrine pathway. Oncogene.

[38] Wang R., Bai Z., Wen X., Du H., Zhou L., Tang Z., Yang Z., Ma W. (2019). MiR-152-3p regulates cell proliferation, invasion and extracellular matrix expression through by targeting FOXF1 in keloid fibroblasts. Life Sci..

[39] Li M., Ren C.-X., Zhang J.-M., Xin X.-Y., Hua T., Wang H.-B. (2018). The Effects of miR-195-5p/MMP14 on Proliferation and Invasion of Cervical Carcinoma Cells Through TNF Signaling Pathway Based on Bioinformatics Analysis of Microarray Profiling. Cell Physiol. Biochem..

[40] Sun Y., Liu W.-Z., Liu T., Feng X., Yang N., Zhou H.-F. (2015). Signaling pathway of MAPK/ERK in cell proliferation, differentiation, migration, senescence and apoptosis. J. Recept. Signal Transduct. Res..

[41] Huang Z., Gao H., Qing L., Wang B., He C., Luo N., Lu C., Fan S., Gu P., Zhao H. (2022). A long noncoding RNA GTF2IRD2P1 suppresses cell proliferation in bladder cancer by inhibiting the Wnt/beta-catenin signaling pathway. PeerJ.

[42] Chuong C.-M., Patel N., Lin J., Jung H.-S., Widelitz R.B. (2000). Sonic hedgehog signaling pathway in vertebrate epithelial appendage morphogenesis: Perspectives in development and evolution. Cell Mol. Life Sci..

[43] Andl T., Reddy S.T., Gaddapara T., Millar S.E. (2002). WNT signals are required for the initiation of hair follicle development. Dev. Cell.

[44] Zhou Y., Xu J., Luo H., Meng X., Chen M., Zhu D. (2022). Wnt signaling pathway in cancer immunotherapy. Cancer Lett..

[45] Zhang Y., Li F., Shi Y., Zhang T., Wang X. (2022). Comprehensive Transcriptome Analysis of Hair Follicle Morphogenesis Reveals That lncRNA-H19 Promotes Dermal Papilla Cell Proliferation through the Chi-miR-214-3p/β-Catenin Axis in Cashmere Goats. Int. J. Mol. Sci..

[46] He M., Lv X., Cao X., Yuan Z., Quan K., Getachew T., Mwacharo J.M., Haile A., Li Y., Wang S. (2023). CRABP2 Promotes the Proliferation of Dermal Papilla Cells via the Wnt/β-Catenin Pathway. Animals.

[47] Livak K.J., Schmittgen T.D. (2001). Analysis of relative gene expression data using real-time quantitative PCR and the 2(-Delta Delta C(T)) Method. Methods.

[48] Chen S., Zhou Y., Chen Y., Gu J. (2018). fastp: An ultra-fast all-in-one FASTQ preprocessor. Bioinformatics.

[49] Langmead B., Salzberg S.L. (2012). Fast gapped-read alignment with Bowtie 2. Nat. Methods.

[50] Kim D., Langmead B., Salzberg S.L. (2015). HISAT: A fast spliced aligner with low memory requirements. Nat. Methods.

[51] Love M.I., Huber W., Anders S. (2014). Moderated estimation of fold change and dispersion for RNA-seq data with DESeq2. Genome Biol..

[52] Robinson M.D., McCarthy D.J., Smyth G.K. (2010). EdgeR: A Bioconductor package for differential expression analysis of digital gene expression data. Bioinformatics.

[53] Huang D.W., Sherman B.T., Tan Q., Collins J.R., Alvord W.G., Roayaei J., Stephens R., Baseler M.W., Lane H.C., Lempicki R.A. (2007). The DAVID Gene Functional Classification Tool: A novel biological module-centric algorithm to functionally analyze large gene lists. Genome Biol..

[54] Ashburner M., Ball C.A., Blake J.A., Botstein D., Butler H., Cherry J.M., Davis A.P., Dolinski K., Dwight S.S., Eppig J.T. (2000). Gene ontology: Tool for the unification of biology. Nat. Genet..

[55] Kanehisa M., Goto S. (2000). KEGG: Kyoto encyclopedia of genes and genomes. Nucleic Acids Res..

